# Supplementary material for: Effects of aquatic exercise on arterial stiffness and endothelial function in adults: A systematic review and meta-analyses
Source: PLoS One. 2025 Dec 12;20(12):e0338929. doi: 10.1371/journal.pone.0338929 (PMC12700369; doi:10.1371/journal.pone.0338929)
Supplement: S1 File — (PDF) [file pone.0338929.s001.pdf]

Search terms

PubMed:

(hemodynamics[Title/Abstract] OR cardiovascular[Title/Abstract] OR "vascular stiffness"[Title/Abstract] OR vascular[Title/Abstract] OR endothelium[Title/Abstract] OR arterial[Title/Abstract] OR venous[Title/Abstract] OR "blood pressure"[Title/Abstract] OR hypertension[Title/Abstract] OR "flow-mediated dilation"[Title/Abstract]) AND ("aquatic therapy"[Title/Abstract] OR hydrotherapy[Title/Abstract] OR "aquatic exercise"[Title/Abstract] OR "aquatic exercises"[Title/Abstract] OR "water exercise"[Title/Abstract] OR "water exercises"[Title/Abstract] OR "water-based exercise"[Title/Abstract] OR "water-based exercises"[Title/Abstract] OR "pool exercise"[Title/Abstract] OR "pool exercises"[Title/Abstract] OR "pool therapy"[Title/Abstract] OR balneotherapy[Title/Abstract] OR "water immersion"[Title/Abstract] OR "swimming"[Title/Abstract])

CINAHL Plus with Full Text and SPORTDiscus with full text:

(hemodynamics OR cardiovascular OR "vascular stiffness" OR vascular OR endothelium OR arterial OR venous OR "blood pressure" OR hypertension OR "flow-mediated dilation") AND ("aquatic therapy" OR hydrotherapy OR "aquatic exercise" OR "aquatic exercises" OR "water exercise" OR "water exercises" OR "water-based exercise" OR "water-based exercises" OR "pool exercise" OR "pool exercises" OR "pool therapy" OR balneotherapy OR "water immersion" OR "swimming")
