# Supplementary material for: Effects of aquatic exercise on arterial stiffness and endothelial function in adults: A systematic review and meta-analyses
Source: PLoS One. 2025 Dec 12;20(12):e0338929. doi: 10.1371/journal.pone.0338929 (PMC12700369; doi:10.1371/journal.pone.0338929)
Supplement: S1 Table — (PDF) [file pone.0338929.s002.pdf]

## S1 Table: Adherence and adverse events

| Study | Aquatic Exercise Group | | | Non-Exercise Comparison | | | Land-Exercise Comparison | | |
|---|---|---|---|---|---|---|---|---|---|
| | **Type** | **Adherence %** | **Adverse Events** | **Type** | **Adherence %** | **Adverse Events** | **Type** | **Adherence %** | **Adverse Events** |
| Alkatan et al. 2016 | swimming | 98% | NR | - | - | - | land-based stationary bicycling | 97% | NR |
| Ha et al. 2018 | aquarobics | NR | NR | Usual routine | NR | NR | - | - | - |
| Haynes et al. 2021 | water-based walking | 79% | NR | Usual routine; education | NR | | land-based walking | 83% | NR |
| Kim et al. 2018 | aquarobics | NR | NR | Usual routine | NR | NR | land-based exercise | NR | NR |
| Klonizakis et al. 2023 | swimming | 93% (2x/wk) 84% (3x/wk) | No adverse events | Usual routine | NR | No adverse events | - | - | - |
| Lee et al. 2018 | water-based treadmill + physical therapy | NR | No adverse events | - | - | - | land-based ergometer + physical therapy | NR | No adverse events |
| Nualnim et al. 2012 | swimming | 99% | NR | - | - | - | land-based relaxation exercise + stretching | NR | NR |
| Park et al. 2019 | water-based walking | 84% | No adverse events | non-exercise activities | NR | NR | - | - | - |
| Park et al. 2020 | water-based walking | 88% | No adverse events | - | - | - | land-based treadmill walking | 81% | No adverse events |
| Ploydang et al. 2023 | water-based Nordic walking | 100% | NR | Usual routine | NR | NR | - | - | - |
| Scheer et al. 2020 | water-based circuit training | NR | No adverse events | Usual routine | NR | NR | - | - | - |
| Scheer et al. 2023 | water-based aerobic exercises | NR | No adverse events | Usual routine | NR | NR | land-based aerobic + resistance exercise | | 1 event – tachycardia |
| Sherlock et al. 2014 | water-based aerobic exercise | NR | NR | Usual routine | NR | NR | - | - | - |
| Son et al. 2024 | water-based water walking | NR | NR | Usual routine | NR | NR | - | - | - |
| Suntraluck et al. 2017 | water-based bicycle training | NR | NR | - | - | - | land-based stationary bicycling | NR | NR |
| Vasić et al. 2019 | water-based aerobic exercise + calisthenics | NR | No adverse events | refrain from exercise | NR | NR | land-based aerobic + calisthenics | | No adverse events |
| Wong et al. 2019 | swimming | NR | No adverse events | Usual routine | NR | NR | - | - | - |
| Xin et al. 2024 | HIIT water-based running | >95% | NR | - | - | - | HIIT land-based running | >95% | NR |

NR: not reported; (-) indicates the comparison group was not included in the study
