# Supplementary material for: Effects of aquatic exercise on arterial stiffness and endothelial function in adults: A systematic review and meta-analyses
Source: PLoS One. 2025 Dec 12;20(12):e0338929. doi: 10.1371/journal.pone.0338929 (PMC12700369; doi:10.1371/journal.pone.0338929)
Supplement: S2 Table — (PDF) [file pone.0338929.s003.pdf]

S2 Table: Revised Cochrane risk of bias tool for randomized trials (RoB 2) - Signalling questions

**Studies**	**Signaling Questions**																											
	1. Bias from randomization process			2. Bias due to deviations from intended interventions													3. Bias due to missing outcome data				4. Bias in the measurement of the outcome					5. Bias in the selection of the reported result		
				part a - effect of assignment to intervention,							part b - effect of adhering to intervention																	
	1.1	1.2	1.3	2.1a	2.2a	2.3a	2.4a	2.5a	2.6a	2.7a	2.1b	2.2b	2.3b	2.4b	2.5b	2.6b	3.1	3.2	3.3	3.4	4.1	4.2	4.3	4.4	4.5	5.1	5.2	5.3
Alkatan et al. 2016	NI	NI	N	Y	Y	N	-	-	Y	-	Y	Y	PY	PN	PN	-	PY	-	-	-	N	N	NI	PY	PY	Y	PN	NP
Ha et al. 2018	NI	NI	N	Y	Y	N	-	-	Y	-	Y	Y	PY	PN	PN	-	Y	-	-	-	N	N	NI	NP	-	NI	PN	NP
Haynes et al. 2021	NI	NI	N	Y	Y	N	-	-	N	PN	Y	Y	PY	PN	PN	-	N	PN	PN	-	N	N	N	-	-	Y	PN	NP
Kim et al. 2018	PY	NI	N	Y	Y	PN	-	-	PN	PY	Y	Y	PY	PN	PY	N	N	PN	NI	NI	N	N	NI	NP	-	NI	PN	NP
Klonizakis et al. 2023	Y	Y	N	Y	Y	N	-	-	PY	PN	Y	Y	PY	PN	PY	N	PN	PN	NI	PN	N	N	N	-	-	NI	PN	PY
Lee et al. 2018	Y	Y	N	Y	Y	N	-	-	PN	PY	Y	Y	PY	PN	PY	N	PN	PN	NI	PN	N	N	N	-	-	NI	PN	PN
Nualnim et al. 2012	N	NI	N	Y	Y	Y	NI	NI	NI	NI	Y	Y	PY	PN	NI	NI	NI	NI	NI	NI	N	N	NI	PY	PY	NI	PN	PN
Park et al. 2019	Y	NI	N	Y	Y	N	-	-	Y	-	Y	Y	PY	PN	PN	-	PN	PN	PN	-	N	N	N	-	-	Y	PN	PN
Park et al. 2020	Y	NI	N	Y	Y	N	-	-	Y	-	Y	Y	PY	PN	PN	-	PN	PN	PN	-	N	N	N	-	-	Y	PN	PN
Ploydang et al. 2023	PY	NI	N	Y	Y	N	-	-	Y	-	Y	Y	PY	PN	NI	-	PY	-	-	-	N	N	NI	PY	PY	NI	PN	PN
Scheer et al. 2020	N	NI	N	Y	Y	Y	PY	N	PN	PN	Y	Y	PY	PN	PY	N	N	PN	PY	NI	N	N	N	-	-	NI	PN	PN
Scheer et al. 2023	PY	Y	N	Y	Y	PN	-	-	PY	-	Y	Y	NI	PN	PY	PN	PN	PN	PY	PY	N	N	N	-	-	Y	PN	PN
Sherlock et al. 2014	NI	NI	N	Y	Y	PN	-	-	PN	PY	Y	Y	Y	PN	PY	PN	PN	PN	PY	PN	N	N	NI	PY	PY	NI	PN	PN
Son et al. 2024	NI	NI	PN	Y	Y	PY	PN	-	PN	PY	Y	Y	NI	PN	PY	PN	PN	PN	PY	PY	N	N	NI	PY	PY	N	PN	PN
Suntraluck et al. 2017	PY	NI	PN	Y	Y	N	-	-	PN	PN	Y	Y	PY	PN	PY	N	PN	PN	PY	PN	N	N	NI	PY	PY	NI	PN	PN
Vasić et al. 2019	PY	Y	PN	Y	Y	N	-	-	PN	PN	Y	Y	PY	PN	PN	-	PY	-	-	-	N	N	NI	PY	PY	Y	PN	PN
Wong et al. 2018	Y	NI	N	Y	Y	N	-	-	PN	PN	Y	Y	PY	PN	PN	-	PY	-	-	-	N	N	NI	NP	-	PN	PN	PN
Xin et al. 2024	Y	NI	PN	Y	Y	N	-	-	PN	PY	Y	Y	PY	PN	PN	-	PN	PN	PY	PN	N	N	NI	PY	PY	NI	PN	PN

Y: yes; N: no; PY: probably yes; PN: probably no; NI: no information; (-) indicates question did not need to be answered: red color flags potential bias, green color favors low risk of bias

Revised Cochrane risk-of-bias tool for randomized trials (RoB 2) - Signalling questions

1.1 Was the allocation sequence random?
1.2 Was the allocation sequence concealed until participants were enrolled and assigned to interventions?
1.3 Did baseline differences between intervention groups suggest a problem with the randomization process?
2.1a. Were participants aware of their assigned intervention during the trial?
2.2a Were carers and people delivering the interventions aware of participants assigned intervention during the trial?
2.3a If Y/PY/NI to 2.1a or 2.2a: Were there deviations from the intended intervention that arose because of the trial context?
2.4a If Y/PY to 2.3a: Were these deviations likely to have affected the outcome?
2.5a If Y/PY/NI to 2.4a: Were these deviations from intended intervention balanced between groups?
2.6a Was an appropriate analysis used to estimate the effect of assignment to intervention?
2.7a If N/PN/NI to 2.6a: Was there potential for a substantial impact (on the result) of the failure to analyze participants in the group to which they were randomized?
2.1b. Were participants aware of their assigned intervention during the trial?
2.2b Were carers and people delivering the interventions aware of participants assigned intervention during the trial?

2.3b [If applicable:] If Y/PY/NI to 2.1 or 2.2: Were important non-protocol interventions balanced across intervention groups?
2.4b [If applicable:] Were there failures in implementing the intervention that could have affected the outcome?
2.5b [If applicable:] Was there non-adherence to the assigned intervention regimen that could have affected participants' outcomes?
2.6b If N/PN/NI to 2.3, or Y/PY/NI to 2.4 or 2.5: Was an appropriate analysis used to estimate the effect of adhering to the intervention?
3.1 Were data for this outcome available for all, or nearly all, participants randomized?
3.2 If N/PN/NI to 3.1: Is there evidence that the result was not biased by missing outcome data?
3.3 If N/PN to 3.2: Could missingness in the outcome depend on its true value?
3.4 If Y/PY/NI to 3.3: Is it likely that missingness in the outcome depended on its true value?
4.1 Was the method of measuring the outcome inappropriate?
4.2 Could measurement or ascertainment of the outcome have differed between intervention groups?
4.3 If N/PN/NI to 4.1 and 4.2: Were outcome assessors aware of the intervention received by study participants?
4.4 If Y/PY/NI to 4.3: Could assessment of the outcome have been influenced by knowledge of intervention received?
4.5 If Y/PY/NI to 4.4: Is it likely that assessment of the outcome was influenced by knowledge of intervention received?
5.1 Were the data that produced this result analyzed in accordance with a pre-specified analysis plan that was finalized before unblinded outcome data were available for analysis?
5.2 Is the numerical result being assessed likely to have been selected, on the basis of the results, from multiple eligible outcome measurements within the outcome domain?
5.3 Is the numerical result being assessed likely to have been selected, on the basis of the results, from multiple eligible analyses of the data?
