# Supplementary material for: Effects of aquatic exercise on arterial stiffness and endothelial function in adults: A systematic review and meta-analyses
Source: PLoS One. 2025 Dec 12;20(12):e0338929. doi: 10.1371/journal.pone.0338929 (PMC12700369; doi:10.1371/journal.pone.0338929)
Supplement: Attachment — Submitted filename: Response to Reviewers.docx [file pone.0338929.s005.docx]

**Rebuttal letter with point-by-point response to reviewers’ comments**

We would like to thank the reviewers and the editor for their time and effort in evaluating our present manuscript (PONE-D-25-37368) entitled “Effects of aquatic exercise on arterial stiffness and endothelial function in adults: A systematic review and meta analyses”. We have modified the manuscript carefully according to the reviewers’ comments and journal requirements (shown in blue below). As instructed by the Journal, the revised sections are visible using Microsoft Word’s Track Changes feature. Line numbers in this point-by-point response refer to the corresponding lines in the manuscript with tracked changes.

**Journal Requirements:**

1. Please ensure that your manuscript meets PLOS ONE's style requirements, including those for file naming. The PLOS ONE style templates can be found at <https://journals.plos.org/plosone/s/file?id=wjVg/PLOSOne_formatting_sample_main_body.pdf>and <https://journals.plos.org/plosone/s/file?id=ba62/PLOSOne_formatting_sample_title_authors_affiliations.pdf>

We have revised the manuscript to ensure that it complies with PLOS ONE’s style requirements, including the file name conventions.

2. In the online submission form, you indicated that the data underlying the results presented in the study are available from the corresponding author ([edunlap@utexas.edu](mailto:edunlap@utexas.edu)).
All PLOS journals now require all data underlying the findings described in their manuscript to be freely available to other researchers, either 1. In a public repository, 2. Within the manuscript itself, or 3. Uploaded as supplementary information.
This policy applies to all data except where public deposition would breach compliance with the protocol approved by your research ethics board. If your data cannot be made publicly available for ethical or legal reasons (e.g., public availability would compromise patient privacy), please explain your reasons on resubmission and your exemption request will be escalated for approval.

All data underlying the research findings described in our manuscript are within the manuscript or in the supporting information.

3. When completing the data availability statement of the submission form, you indicated that you will make your data available on acceptance. We strongly recommend all authors decide on a data sharing plan before acceptance, as the process can be lengthy and hold up publication timelines. Please note that, though access restrictions are acceptable now, your entire data will need to be made freely accessible if your manuscript is accepted for publication. This policy applies to all data except where public deposition would breach compliance with the protocol approved by your research ethics board. If you are unable to adhere to our open data policy, please kindly revise your statement to explain your reasoning, and we will seek the editor's input on an exemption. Please be assured that, once you have provided your new statement, the assessment of your exemption will not hold up the peer review process.

Again, all data underlying the findings described in our manuscript are within the manuscript or in the supporting information.

4. Please include captions for your Supporting Information files at the end of your manuscript, and update any in-text citations to match accordingly. Please see our Supporting Information guidelines for more information: http://journals.plos.org/plosone/s/supporting-information.

We have added captions for our supporting information at the end of our manuscript. We also separated our supporting information into individual documents and updated all in-text citations to match accordingly.

5. If the reviewer comments include a recommendation to cite specific previously published works, please review and evaluate these publications to determine whether they are relevant and should be cited. There is no requirement to cite these works unless the editor has indicated otherwise.

Thanks for this comment. We have reviewed the feedback and note that no specific citations were recommended.

6. While revising your submission, please upload your figure files to the Preflight Analysis and Conversion Engine (PACE) digital diagnostic tool, [https://pacev2.apexcovantage.com/](https://track.editorialmanager.com/CL0/https:%2F%2Fpacev2.apexcovantage.com%2F/1/010f01989d72054b-ce333fc5-4e77-41e4-8b51-bb6e8c9c801d-000000/4bqqwFEv7ZbViHHK_lGuL659UbBuzUO5VVwfD-eBl50=223). PACE helps ensure that figures meet PLOS requirements. To use PACE, you must first register as a user. Registration is free. Then, login and navigate to the UPLOAD tab, where you will find detailed instructions on how to use the tool. If you encounter any issues or have any questions when using PACE, please email PLOS at [figures@plos.org](mailto:figures@plos.org). Please note that Supporting Information files do not need this step.

We have uploaded all figures into the PACE digital diagnostic tool and reviewed the PACE-adjusted versions to confirm clarity and content.

**Reviewer #1:**

This is a well-conducted systematic review of aquatic exercise interventions on vascular function. The authors address the clinically important topic, but the manuscript would benefit from a more nuanced interpretation of the findings. The review provides moderate-certainty evidence that aquatic exercise improves vascular function, particularly endothelial function, more than no exercise and possibly more than land-based exercise. However, conclusions should be toned down as there are methodological limitations, high heterogeneity, and risk of bias in the included studies. However, this manuscript contributes valuable insight into how aquatic exercise may influence vascular health, particularly in older adults or clinical populations.

Thank you for your thoughtful feedback and the opportunity to improve this review. As described below, we have made extensive revisions on the manuscript.

**MAJOR COMMENTS:**
The pooled analysis for PWV versus non-exercise has an I2 of 98%, which is extremely high. Moderate GRADE ratings were given even when risk of bias and heterogeneity were both high. The justification for not downgrading for inconsistency is debatable. The argument that sensitivity analysis "rescues" the finding is not sufficient to ignore heterogeneity entirely. While a sensitivity analysis helps explain some of this, the clinical and methodological sources of heterogeneity (e.g., water temperature, exercise modality, protocol duration) need clearer exploration. Does the heterogeneity reduce the confidence in the generalizability of the findings? Maybe a subgroup analysis is needed.

The impact of measurement variability on effect sizes and heterogeneity needs more discussion. Studies using peripheral versus central pathways are not equivalent. Did the authors adjust the meta-analysis to account for which segment of the vasculature was measured? For example, did they analyze central PWV separately from peripheral PWV? Or did they combine them all together?

Thanks for your thoughtful comments regarding sources of heterogeneity in the comparison of aquatic versus non-exercise interventions on PWV and your comments on the PWV measurement type variability (peripheral vs central pathways). To address this, we reclassified our initial sensitivity analysis (comparing PWV measurement types—central/systemic vs peripheral PWV) as a subgroup analysis. (see Figure 2a and Table 2 and Results lines 338-345, 364-370) We believe this approach is methodologically appropriate and addresses the reviewer’s concerns, as these PWV types represent distinct physiological constructs. Notably, heterogeneity was substantially reduced (I² = 0%) within the central/systemic PWV subgroup, suggesting measurement type partly explains between-study variability.

For consistency, we applied the same subgrouping approach to the aquatic versus land-based exercise comparison even though heterogeneity was low (see Figure 3a and Table 2 and Results lines 347-356, 373-380). Only one study in this subgroup used a peripheral PWV measure, which we reported separately rather than pooled. In the GRADE Summary of Findings table (Table 2), PWV outcomes are presented by measurement type, improving transparency by clarifying that peripheral PWV measures are associated with lower certainty—due to both high heterogeneity in the aquatic versus control comparison and limited data in the aquatic versus land-based comparison.

We acknowledge that additional clinical and methodological factors may contribute to residual heterogeneity, which could lower confidence in the peripheral arterial stiffness findings and highlight areas of focus for future research to build on the current level of evidence. These statements have been inserted in the revised manuscript (Discussion lines 548-550 and Conclusion lines 581-582).

The authors should explore more granular sources of heterogeneity, such as population characteristics, exercise modality (e.g., aqua-aerobics vs. swimming), and vascular segment measured. Consider re-running sensitivity analyses excluding high-risk studies altogether to see if main conclusions still hold.

We appreciate the reviewer’s suggestion to explore more granular sources of heterogeneity. To address this, we conducted several additional investigations for the purpose of reviewer evaluation, they can be found at the end of this letter. These analyses included subgroups by sex, health status (healthy vs. disease), aquatic intervention type (swimming vs. walking vs aquarobics), and intervention duration (≥11 weeks vs. 11 weeks). Sensitivity analyses excluding high-risk studies to test robustness were also performed and can be found at the end of this letter. In summary, the subgroupings explored did not meaningfully reduce heterogeneity. As such, these additional analyses will not be included in the final manuscript but are provided at the end of this letter for transparency and the consideration of the reviewer.

We acknowledge that some residual heterogeneity remains unexplained which lowers the level of certainty for some findings. As mentioned earlier, we have revised the manuscript with this clarification and highlighted areas of focus for future research to build on the current level of evidence (discussion lines 519-532, 538-542, and conclusion lines 581-582).

The conclusions rely heavily on studies with high risk of bias, yet this issue is underemphasized in the discussion. The authors should more explicitly discuss how these limitations might have distorted the findings, particularly for FMD. Tone down conclusions of “superior” effect and emphasize suggestive rather than definitive evidence, in line with GRADE and CI boundaries.

Thank you for this important observation. We agree that the conclusions, particularly regarding FMD, may have overstated given the low level of certainty. In response, we have revised the results and discussion to more clearly acknowledge the high proportion of studies at high risk of bias or high heterogeneity contributing to the pooled estimate (discussion lines 548-550). We explicitly state that these methodological limitations may have inflated the observed effects, and we highlight this as a key limitation affecting confidence in the findings.

Additionally, we have revised our wording throughout the abstract, discussion and conclusion sections to reflect **suggestive rather than definitive** evidence, in accordance with the GRADE assessment and the wide confidence intervals. Phrases such as “superior effect” have been replaced with more cautious language (e.g., “suggestive of a benefit” or “may be more effective”), consistent with the level of certainty and the available data (see lines 47-52, 71-78, 433-43, 445).

**MINOR COMMENTS:**
In the abstract, try to include exact values: SMDs, CIs, I² for PWV and FMD outcomes, and clearly highlight the main conclusion.

The abstract has been revised to include findings from the PWV subgroups along with all SMDs, CIs, I^2^, for all findings (PWV subgroups and FMD). See lines 35-46.

In Table 1, water depth is missing for several interventions. Please clarify if the information was not reported by the study or if it was omitted here.

Thank you for pointing this out. The column in Table 1 represents both pool temperature and depth. For three studies that originally reported temperature but not depth, we have now added “NR Depth” to indicate that water depth was not reported. This change clarifies the missing information. For studies where neither temperature nor depth was reported, the column remains labeled as “NR.”

For consistency and clarity, also provide mean ± SD (if available) for age so readers can compare across studies. A range alone is less informative.

We appreciate your attention to detail. For Suntraluck et al., only a range was provided in the original publication, which is why it appears that way in the Table 1. To improved clarity, we revised the table caption to state: “Data presented as means ± SD unless otherwise stated”.

Page 20: “This increased blood flow generates greater flow throughout the vascular system.” This sentence is redundant. Consider revising to: “This increased blood flow enhances shear stress throughout the vascular system.”

Thanks for the suggestion to reduce redundancy. This has been revised as suggested (line – 452).

Page 21: “This mechanistic explanation is consistent with our findings of superior endothelial function...” Provide a specific citation to support the mechanistic claim or refer back to a particular figure/table in your analysis.

Thank you for the comment. We have revised this section (lines 457-476).

Page 22: “The water walking exercise may allow for more social engagement...”
Consider acknowledging this as a hypothesis or anecdotal observation unless you have qualitative data to support it.

A sentence has been added to clarify that we do not have direct data to confirm this to acknowledge this as a hypothesis (lines 505-607)

**Reviewer #2:**

The manuscript has strong potential, but it requires improvements in multiple sections, particularly in clarity, transparency of reporting, and adherence to systematic review best practices.

Thanks for the complimentary comment and the opportunity to improve this manuscript with your insightful feedback.

**Abstract**
Include more specific details about the search period (e.g., “from database inception to April 16, 2025”).

The specific details about the search period have been added to the abstract (line 26)

The conclusion should mention the heterogeneity observed, as this affects the interpretation of the meta-analysis results.

Thank you for this suggestion. We agree that heterogeneity is an important factor influencing interpretation of our findings. In our revision, we addressed this in two ways: (1) by conducting subgroup analyses, which helped to explain some of the observed heterogeneity, and (2) by incorporating heterogeneity into our certainty of evidence assessments using GRADE, where some outcomes were rated as low certainty partly due to this issue. Given these points, we added specific language in the conclusion to indicate the level of certainty. We feel that this addressed the main concern about the impact of heterogeneity (lines 47-52).

**Methods**
Line 138-147: The inclusion criteria should be more detailed. Specify the participants characteristics and types of interventions explicitly.

Details have been added about participant characteristics and types of interventions. (lines 151-159)

Line 179: Provide more detail on the meta-analysis approach, particularly the rationale for using the random-effects model.

We have clarified the meta-analysis approach, including the rationale for selection of SMD vs mean differences and selection of random-effects model in the revised Methods section. Specifically, we note that SMD were selected rather than mean differences because there was considerable variability in outcome distributions across studies (lines 180-182) Additionally, the random-effects model was chosen because we anticipated true variation in effect sizes across studies due to differences in participant populations, intervention protocols (e.g., exercise modality, duration, and intensity), and vascular measurement techniques (lines 194-196).

**Results**
Page 15, first paragraph: Describe the characteristics of the included studies in more detail. For example, include information on the sample, sample size range, and the settings of the interventions.

We have revised the results section to provide more detail of the study characteristics. This information is now presented in the revised results section (lines 301-306) and summarized in Table 1 for clarity.

Page 15, first paragraph: Please rephrase the sentence ‘Water walking 18,20,27,28,30,39 and combined walking-aerobic exercises,19,35–37,40,41 with six studies each’ for clarity.

This has been adjusted for clarity and now reads “Aquatic exercise interventions varied across studies. Six studies implemented water walking protocols,^18,20,27,28,30,39^ while another six used combined walking and aerobic exercises ^19,35–37,40,41^” (Lines 307-308).

Page 16, second paragraph: Address the potential impact of heterogeneity. The high I² values reported (e.g., I²=98%) indicate substantial heterogeneity, which should be explored further in the text.

Thank you for this valuable comment. We originally conducted sensitivity analyses to assess the robustness of findings but, in light of the reviewer’s observation regarding the high heterogeneity (e.g., I² = 98% in PWV outcomes), we revised our approach and conducted subgroup analyses to explore its potential sources. Specifically, we stratified studies by PWV measurement type (central/systemic vs. peripheral). This reclassification substantially reduced heterogeneity in the central/systemic PWV subgroup (I² = 0%) while heterogeneity remained high in the peripheral subgroup (I² = 98%), suggesting measurement type as a contributing factor. Although heterogeneity was low in the aquatic vs. land-based comparisons (I² = 0%), we conducted parallel subgroup analyses for consistency. These revisions have been added to the revised Results section and incorporated into the GRADE assessments (lines 338-345, 347-356, 364-370, 373-380).

**Discussion**
Provide a more detailed discussion of the potential sources of heterogeneity observed in the meta-analysis. Discuss factors such as differences in sample characteristics, intervention types, and dosage.

We agree that a deeper exploration of heterogeneity would strengthen the manuscript. In response, we have expanded the discussion to more thoroughly address the potential sources of heterogeneity in our meta-analyses. (lines 464-471, 519-532)

Suggest clearer implications for clinical practice and future research. For example, recommend specific areas where further high-quality RCTs are needed.

To address this comment, we revised the discussion to clearer state the implications for clinical practice and future research. Specific areas where further high-quality research is needed are outlined based on the RoB2 findings that identified sources of high risk of bias in the included studies (lines 538-542, 548-550, 553-558).

**Conclusion**
Emphasize the need for better-designed RCTs that address the limitations identified in this review, particularly the issue of inadequate demographic factors and heterogeneity.

In the revised conclusion, we incorporated clear directions for future research based on the findings of the ROB2 assessment. We emphasized the need for well-designed randomized controlled trials, particularly in the area of endothelial function, where the current evidence is of low certainty due to high risk of bias and heterogeneity. Specifically, we now state (see lines 580-583)

“Future research should focus on well-designed randomized controlled trials with larger sample sizes, blinded assessors, and strategies for participant retention, in order to strengthen the evidence base and help refine aquatic exercise prescriptions for vascular function benefits”.

**Figures and Tables**

Strengths: The figures and tables are informative and well-organized.

Thank you!

Improvements: Ensure all acronyms are defined in the table legends in Table 2 (e.g., SMD, PWV, FMD, RCT).

Thanks for the comment. All acronyms have now been defined in the legends in Table 2 (lines 422-423).

The forest plots should include a clearer indication of the confidence intervals and heterogeneity statistics (I² values).

Thank you for your comment. The forest plots were generated using RevMan, which automatically includes confidence intervals and heterogeneity statistics (I² values) for each analysis. These are displayed within each figure and conform to standard reporting formats for meta-analyses. As the figures are software-generated, our ability to alter their formatting is limited, but we have reviewed them carefully to ensure that these elements are visible and clearly presented.

**References**
Ensure all references are formatted consistently according to PLOS One’s citation style, particularly regarding journal titles.

Thanks for pointing this out. All references, including journal titles, have been updated to comply with PLOS ONE guidelines. DOIs were added manually, and changes are not visible in Track Changes due to Word issues.

**Reviewer #3:**

Benefits in vascular function (endothelial function and arterial stiffness) with aquatic exercise compared to land-based exercise have been reported previously. The current manuscript confirms those benefits but, as written, does not present a clearly novel finding for long-term vascular health in older adults: when exercise is performed consistently, aquatic and land-based modalities appear similarly effective.

Thank you for this comment. It should be noted that most of the previous studies suffer from small sample sizes. As such it is difficult to draw definitive conclusions without meta-analysis. While we agree that vascular benefits of aquatic and land-based exercise have been reported previously, our study adds novelty in several important ways. First, we provided a quantitative synthesis of arterial stiffness and endothelial function outcomes, which to our knowledge has not been systematically evaluated before. Second, our findings suggest that aquatic exercise may lead to greater improvements in endothelial function compared to land-based exercise, although the certainty of this evidence is low. Finally, we applied the GRADE framework to evaluate the certainty of evidence, which extends prior findings by clarifying the limitations of the current literature. Together, these contributions extend prior work by confirming that aquatic and land-based exercise appear similarly effective overall for arterial stiffness, while also highlighting a potential added benefit of aquatic exercise for endothelial function. Importantly, our findings identify key areas for future research to strengthen the certainty of evidence and enhance understanding of vascular function outcomes after aquatic exercise.

To add value and clarify effect modifiers, the authors should consider subgroup analyses stratified by age, sex, and disease status (see recommended subgroup scheme below). These comparisons would help reveal whether aquatic exercise confers different benefits in specific populations (e.g., older women, younger men with diabetes) and would strengthen the manuscript’s contribution.

**Recommended subgrouping scheme** (for analysis & figures)
Perform subgroup analyses for each outcome (FMD, pulse wave velocity/arterial stiffness, NO metabolites, etc.) using the following strata:
Older men — healthy (aquatic vs land vs control)
Older men — disease (e.g., diabetes, established CVD
Younger men — healthy (aquatic vs land vs control)
Younger men — disease (e.g., diabetes, established CVD)
Older women — healthy (aquatic vs land vs control)
Older women — disease (e.g., diabetes, established CVD)
Younger women — healthy (aquatic vs land vs control)
Younger women — disease (e.g., diabetes, established CVD)

Thank you for this insightful suggestion. We agree that sex, age, and health status could influence vascular function outcomes, and we explored these subgroupings. However, most of the included studies enrolled mixed-sex populations without stratified reporting, and the available data were insufficient to calculate sex-specific effect estimates. Participants were generally older (range was 52–76 years in the included studies), with no younger adult populations, and no studies met criteria for older men with disease.

Although a few studies (e.g., Xin and Kim) fit the healthy older adult subgroup (aquatic vs. land), these were already included in our existing PWV analysis, which showed very low heterogeneity (I² = 0%). Similarly, while we generated subgroup plots for older women (Ha—healthy, Kim—healthy, Son—obese, Wong—hypertension), these did not substantially reduce heterogeneity. We have included the potential subgroupings for transparency at the end of this document, but as they produced only minor changes with heterogeneity, we did not retain them in the manuscript.

We recommend only the subgrouping by PWV type, as reflected in the current manuscript revisions. This was the only subgroup that substantially reduced heterogeneity in the aquatic vs control arterial stiffness analysis (I^2^ = 98% with all PWV then central/systemic PWV subgroup reduced to I^2^ = 0%).

Finally, the Risk-of-Bias table is not sufficiently clear. The authors should expand and clarify the RoB presentation so readers can assess study quality at a glance.

We appreciate this suggestion. To improve clarity and transparency, we expanded the Risk-of-Bias presentation by providing detailed information on the grading for each Cochrane RoB2 signalling question. This information is now included in the supplementary material (lines 428-430, Table S2), allowing readers to assess study quality more easily at a glance.

**Exploratory Subgroup Analyses**

**Subgroups that were selected and are included in the revised manuscript:**

Aquatic vs. Control and Aquatic vs. Land: PWV

- Subgroup by measurement pathway (central/systemic PWV vs peripheral PWV). Subgroups were selected as the best subgroup to reduce the heterogeneity for aquatic vs control and then matched subgrouping for aquatic vs land for consistency. (Figures 2a and 3a)

Aquatic vs. Control and Aquatic vs. Land: FMD

- Subgroup by adults with and without a reported medical condition. Subgroups were selected as the best subgroup to reduce the heterogeneity especially for aquatic vs land and then matched subgrouping for aquatic vs control for consistency. (Figures 3b and 4b)

**Subgroups that were NOT selected but shared here for transparency to reviewers**

Note - There was only one forest plot that contained more than one study with exclusively female participant.

Aquatic vs Control: PWV – subgroups by sex and ‘healthy’ or disease state

This doesn’t add much value because the subgroups all have I>90%

Aquatic vs Control: PWV – subgroups of women only vs men + women

This doesn’t add much value because the subgroups all have I>90%

Aquatic vs Control: PWV – subgroups of adults with vs without reported medical condition

This doesn’t add much value because the subgroups all have similar I^2^. The subgroup by PWV type (central/systemic vs peripheral) is a better subgroup for PWV.

Aquatic vs Control: PWV – subgroups by exercise type

This doesn’t add much value because the subgroups all have similar I^2^.

Aquatic vs Control: FMD - subgroups by exercise type

This doesn’t add much value because the subgroups all have similar I^2^.

Aquatic vs Land: FMD - subgroups by exercise type

This doesn’t add much value because 3 of the subgroups only have one study

Note for clarification about the forest plots with subgroups by exercise duration.

- The duration selected was ≥8 weeks based on information from meta-analysis which indicates this is the lower range of duration to expect endothelial function changes (You, Qiuping et al. “Effects of Different Intensities and Durations of Aerobic Exercise on Vascular Endothelial Function in Middle-Aged and Elderly People: A Meta-Analysis.” *Frontiers in physiology* 12 (2022): 803102. Web.)
- Aquatic vs Control: PWV –no subgroup needed because all studies were ≥8 weeks in duration

Aquatic vs Control: FMD – subgroup by exercise duration

This doesn’t add much value because the second subgroup only has one study. Also, the first subgroup does not have a large reduction in I^2^.

Aquatic vs Land: FMD - – subgroup by exercise duration

This doesn’t add much value because the second subgroup only has one study. Also, the first subgroup does not have a reduction in I^2^.

**Sensitivity investigation (taking out all high-risk studies)**

Aquatic vs. control: When restricting to studies without high risk of bias the findings are no longer significant, and heterogeneity is 100%. However, this comes only from studies evaluating peripheral arterial stiffness. Two studies show improvement in peripheral PWV while one does not. Since these studies are not low risk but rather ‘some risk of bias’ and has very high heterogeneity, this analysis adds limited value. In the revised manuscript, we clarified that the certainty of the evidence is low for peripheral PWV (due to heterogeneity and risk of bias), which we believe is sufficient. Including this sensitivity analysis in the manuscript would make it unnecessarily confusing.

The last three sensitivity analyses are not informative, as only one study remained in each that was not at high risk of bias.
