# Supplementary material for: Effects of aquatic exercise on arterial stiffness and endothelial function in adults: A systematic review and meta-analyses
Source: PLoS One. 2025 Dec 12;20(12):e0338929. doi: 10.1371/journal.pone.0338929 (PMC12700369; doi:10.1371/journal.pone.0338929)
Supplement: Attachment — Submitted filename: Response to Reviewers (2).docx [file pone.0338929.s006.docx]

**Rebuttal letter with point-by-point response to reviewer comments**

We would like to express our sincere appreciation to the reviewers and the editor for their time and effort in evaluating our manuscript (PONE-D-25-37368) entitled “Effects of aquatic exercise on arterial stiffness and endothelial function in adults: A systematic review and meta-analyses.” The manuscript has been thoroughly revised in response to the reviewers’ insightful comments (indicated in blue below). Line numbers cited in this point-by-point response correspond to those in the revised version with tracked changes.

Journal Requirements:

1. Please review your reference list to ensure that it is complete and correct. If you have cited papers that have been retracted, please include the rationale for doing so in the manuscript text, or remove these references and replace them with relevant current references. Any changes to the reference list should be mentioned in the rebuttal letter that accompanies your revised manuscript. If you need to cite a retracted article, indicate the article’s retracted status in the References list and also include a citation and full reference for the retraction notice.

We have reviewed our reference list and can confirm that none of the cited papers have been retracted.

Reviewer #1: The authors have strengthened the manuscript since the prior round, especially adjusting conclusions around FMD in line with GRADE. However, some comments still remain:

Thank you for your insightful feedback and the opportunity to improve this review.

2. The current “central/systemic” subgroup conflates cfPWV (central) with baPWV (systemic). These are related but distinct constructs and should not be pooled together. Please re-run and report PWV in three strata: central (cfPWV), systemic (baPWV), and peripheral segmental (e.g., crPWV, cbPWV, faPWV).

Thanks for bringing it to our attention. We re-ran the analysis using three PWV subgroups (systemic, peripheral, and central). This resulted in a non-significant difference between subgroups (p = 0.15); therefore, we retained the overall PWV summary in the GRADE table, rated as low confidence due to high overall risk of bias and substantial heterogeneity. We elaborated on the subgroup findings in the Discussion, noting that the systemic and peripheral subgroups demonstrated the significantly positive effects favoring aquatic exercise, while central PWV showed a trend towards improvement with the aquatic group that did not reach statistical significance. These updates are reflected in Figures 2 and 3, Table 2, and in the corresponding highlighted revisions (see lines 35-39, 43-45, 67-69, 338-342, 345-346, 367-372, and 415-417, 420-426, 429-432, 519-530, 574) in the manuscript.

3. The SMD for peripheral PWV stands out (-3.76)? Can we have another look at the SDs from each study – one study can make the effect size inflated. Were medians or standard errors converted correctly? Maybe a “leave-one-out” analysis is needed here… especially with high heterogeneity…

We appreciate the reviewer’s comment regarding the large effect observed for peripheral PWV in the aquatic versus control analysis. We carefully re-examined the SDs from the studies contributing to the peripheral PWV outcome and confirmed that all values are correct. Each of these studies reported mean/SD. We used a conversion formula to estimate the within-group SD of change scores: SD_diff = √(SD_pre² + SD_post² - 2r × SD_pre × SD_post), assuming a correlation coefficient (r) of 0.5 between pre- and post-intervention measures (from Higgins et al. 2020: reference #26). This formula is listed on lines 180-183.

Details for each study are listed below for transparency.

To further assess the influence of individual studies, we conducted a leave-one-out sensitivity analysis by removing the Wong study, which had the largest effect size. The overall results remained significant, supporting the robustness of the findings. The sensitivity analyses is presented below for reference (run with SMD and MD)

4. Why use SMD for PWV? If the SDs are very different between studies, the SMD values can become misleading… maybe use mean differences in m/s

We considered the reviewer’s suggestion of using mean difference in m/s vs. standardized mean differences (SMDs) for pulse wave velocity (PWV). While we agree that SMDs can be influenced by variability in standard deviations across studies, they were chosen because the included studies assessed PWV at different arterial segments—central, systemic, and peripheral—with distinct baseline values and physiological ranges. Although all PWV outcomes are reported in m/s, absolute values are not directly comparable across segments. Pooling mean differences in m/s would therefore be inappropriate. Using SMDs allowed standardization across measurement sites, enabling synthesis of overall effects while accounting for between-segment variability.

To address the reviewer’s concern, we conducted subgroup analyses by PWV segment (central, systemic, peripheral) using mean differences (MDs) within each subgroup. The direction and magnitude of effects were similar across subgroups, supporting the robustness of our findings. These results are shown below but are not included in the manuscript for conciseness.

Finally, we clarified our rationale in the manuscript. See lines 176-180.: “SMDs were used rather than mean differences to account for methodological variability across studies. PWV was measured at different arterial segments (central, peripheral, or systemic), and while most studies assessed brachial FMD, one study measured popliteal FMD. Using SMDs allowed standardization of effect sizes across these different measurement sites.”

5. It will be helpful to explicitly state that the 95%CI for aquatic vs. land spans from trivial to moderate, justifying the low-certainty grade

Thank you. We agree that this will help improve transparency. We added language to explicitly state that the 95% CI for aquatic vs land spans from trivial to moderate, justifying the low-certainty grade. See lines 425-426 (below Table 2). “^c^Downgrade due to considerable heterogeneity across trials and the 95%CI of the SMD spans from a trivial to large effect.”

6. Let the reader know that the subgroup analysis with the medical condition group vs. healthy is a between study comparison… there were differences in water temperature and depth, etc.

Thanks for this recommendation. This language has been added in the Discussion about the subgroup analyses. See lines 480-484: ”While other factors such as water temperature, depth, and exercise protocols may also have influenced the observed variation, this complexity underscores the need to consider both individual and environmental factors when optimizing aquatic exercise interventions for vascular health.”

7. Testing for publication bias is only meaningful with at least 10 studies…

Thanks for pointing this out. We clarified in the Results section why we did not downgrade for publication bias. See lines 416-417 “Publication bias was not formally assessed due to fewer than 10 studies; therefore, no downgrade was applied, though the possibility of bias cannot be ruled out.”
