# Supplementary material for: Effects of aquatic exercise on arterial stiffness and endothelial function in adults: A systematic review and meta-analyses
Source: PLoS One. 2025 Dec 12;20(12):e0338929. doi: 10.1371/journal.pone.0338929 (PMC12700369; doi:10.1371/journal.pone.0338929)
Supplement: Attachment — Submitted filename: Response to Reviewers (3).docx [file pone.0338929.s007.docx]

**Rebuttal letter with point-by-point response to reviewer comments**

We would like to express our sincere appreciation to the reviewers and the editor for their time and effort in evaluating our manuscript (PONE-D-25-37368) entitled “Effects of aquatic exercise on arterial stiffness and endothelial function in adults: A systematic review and meta-analyses.” The manuscript has been revised in response to the most recent reviewer comments (indicated in blue below). Line numbers cited in this point-by-point response correspond to those in the revised manuscript version with tracked changes (tracked with yellow highlight).

Journal Requirements:

1. Please review your reference list to ensure that it is complete and correct. If you have cited papers that have been retracted, please include the rationale for doing so in the manuscript text, or remove these references and replace them with relevant current references. Any changes to the reference list should be mentioned in the rebuttal letter that accompanies your revised manuscript. If you need to cite a retracted article, indicate the article’s retracted status in the References list and also include a citation and full reference for the retraction notice.

We have reviewed our reference list and can confirm that none of the cited papers have been retracted.

Reviewer #1: 

2. Since the central PWV subgroup had one study with a non-significant effect, please revise the conclusion and abstract to specify that it was systemic PWV that improved vs non-exercise and central PWV remains uncertain.

We thank the reviewer for this suggestion. We have clarified throughout the Abstract, Table 2 (GRADE summary), Discussion, and Conclusion that the observed improvement in PWV reflects changes in systemic and peripheral PWV, whereas evidence for central PWV remains inconclusive due to limited data (one study, non-significant effect). See lines 37-39, 42, 44-45, 434-437, and 585 for these updates.

3. Prediction intervals need to be added to all main meta-analyses. Consider Hartung-Knapp sensitivity for high heterogeneity as well.

Thank you for this recommendation. The Hartung-Knapp 95% prediction intervals have been added for all main meta-analyses. Based on the interpretation of these prediction intervals, we have downgraded the certainty of evidence for the FMD aquatic vs control analysis (prediction interval is -0.73 to 2.55). Corresponding details have been incorporated into Table 2, Methods, and Results. Please see Table 2, and lines 200-202, 338, 349-350, 367-377, 414-416, 422-423, and 424 (Table 2) for these updates

4. Fair point with SMDs, but they are still hard to interpret clinically. Could the authors please add a translation of SMDs into approximate absolute units (% for FMD, m/s for PWV). This could simply be in the discussion.

We appreciate the reviewer’s helpful suggestion. We have added a translation of the SMD into approximate absolute units to aid interpretation in the Discussion. See lines 440-447 for this update.

5. Was there a difference in FMD protocols in these studies? (arterial site, cuff position, occlusion duration)

There was some slight variability in FMD protocols across studies, though less than for PWV measurements. Almost all studies measured brachial artery FMD with the cuff positioned on the forearm and an occlusion duration of 3–5 minutes. The only exception was Suntraluck et al. (2017), which measured popliteal FMD with a 3-minute occlusion. This information has been added to lines 328-329.

6. SMD -0.07 does not mean "similar effects" - more neutral phrasing is needed here (e.g., "did not differ")

Thank you for this comment. We have revised the phrasing to be more neutral. See lines 45-46, 69-70, 436-437, 587-588 for these updates

7. Adverse events were mostly not reported - this has to be better reported

Thanks for pointing this out to help improve transparency. We have clarified the information on adverse event reporting in the Results and Discussion Sections. See lines 396-397 and 524-528 for these updates
