# Supplementary material for: Effects of aquatic exercise on arterial stiffness and endothelial function in adults: A systematic review and meta-analyses
Source: PLoS One. 2025 Dec 12;20(12):e0338929. doi: 10.1371/journal.pone.0338929 (PMC12700369; doi:10.1371/journal.pone.0338929)
Supplement: Attachment — Submitted filename: Response to Reviewers (4).docx [file pone.0338929.s008.docx]

**Rebuttal letter with point-by-point response to reviewer comments**

We would like to express our sincere appreciation to the reviewers and the editor for their time and effort in evaluating our manuscript (PONE-D-25-37368) entitled “Effects of aquatic exercise on arterial stiffness and endothelial function in adults: A systematic review and meta-analyses.” The manuscript has been revised in response to the most recent reviewer comments (indicated in blue below). Line numbers cited in this point-by-point response correspond to those in the revised manuscript version with tracked changes (tracked with yellow highlight).

Journal Requirements:

Please review your reference list to ensure that it is complete and correct. If you have cited papers that have been retracted, please include the rationale for doing so in the manuscript text, or remove these references and replace them with relevant current references. Any changes to the reference list should be mentioned in the rebuttal letter that accompanies your revised manuscript. If you need to cite a retracted article, indicate the article’s retracted status in the References list and also include a citation and full reference for the retraction notice.

We have reviewed our reference list and can confirm that none of the cited papers have been retracted.

Reviewer #1: 

1. Some highly heterogeneous results (e.g., FMD aquatic vs land-based; peripheral PWV) appear in the abstract and conclusion without an explicit statement that heterogeneity limits generalizability.

Abstract (lines 46–47):
“Aquatic exercise may provide greater improvement in endothelial function than land-based exercise, though this is supported by low-certainty evidence.”
Please add at the end:
“…though this is supported by low-certainty evidence, and substantial heterogeneity limits confidence in the generalizability of this finding.”

Thanks for this recommendation to clarify the limits of the findings. This terminology has been added – see lines 48-49.

Discussion (lines 434–437): Please add “However, for both FMD and peripheral PWV, substantial between-study heterogeneity and wide prediction intervals indicate that the effects observed in this meta-analysis may not be consistently reproduced in new, similar studies.”

This terminology has been added – see lines 436-439.

Conclusion (lines 585–588): Please change “…and may be more effective for improving endothelial function, although this latter finding is based on low-certainty evidence.” to “…and may be more effective for improving endothelial function, although this finding is based on low-certainty evidence and is limited by high heterogeneity across included studies.”

Thank you for this recommendation. This language has been added – see lines 597-599.

2. Back-translated SMD... absolute units should be described as rough approximations, not pooled mean differences.

Discussion lines 440–447, where you introduce the approximate “clinical translation.”
After the sentence ending “…comparable to improvements typically reported after land-based aerobic training [45].” add:
“These values represent rough approximations based on back-transformation of pooled standardized effects, rather than pooled mean differences from the included trials, and should therefore be interpreted cautiously.”

Thank you for the recommendation for clarify and cautious interpretation. This language has been added – see lines 450-452.

3. Subgroup (medical vs non-medical) was not pre-registered and other moderators could plausibly influence effects but were unanalyzable.

Methods (after describing subgroup by medical condition), please add: “This subgroup was not pre-specified in the PROSPERO registration but was conducted post-hoc to explore potential sources of heterogeneity observed in the primary analyses.”

Thanks for this recommendation to improve transparency. Language to clarify that the subgroups were not pre-specified has been added to the Methods section – see lines 200-202.

Discussion (lines 487–497), after the sentence ending “…wider margin for physiological adaptation.” please add: “Because this subgroup analysis was exploratory rather than pre-registered, its findings should be interpreted with caution. Other factors such as water temperature, immersion depth, participant age, and exercise modality could not be evaluated due to insufficient or inconsistent reporting across studies, leaving possible confounders unaddressed.”

Thank you for this recommendation. This language has been added to the Discussion – see lines 500-504

4. Some wording in Abstract and Discussion is still slightly stronger than warranted by low-certainty GRADE ratings (though the authors have made some adjustments since the last submission):

Abstract (lines 40–41): “Aquatic exercise improved outcomes versus non-exercise controls…” should be “Aquatic exercise may improve outcomes versus non-exercise controls…”

Thanks for the recommendation to tone down the wording to reflect the low-certainty GRADE rating. See lines 40 and 42.

Discussion (lines 436–437): “Aquatic exercise may additionally lead to greater improvements in endothelial function than land-based exercise…” should be “Aquatic exercise may additionally lead to greater improvements in endothelial function than land-based exercise, although this conclusion remains tentative due to low-certainty evidence and substantial variation across studies.”

Thank you, this adjustment has been made – see lines 440-442.

Discussion (lines 455–458): Don't imply causal certainty when describing hydrostatic pressure.. maybe say “…although these mechanistic interpretations remain speculative and were not directly tested in the included trials.”

Thanks for the suggestion. This language has been added in lines 473-475.

Conclusion (lines 585–588): “Aquatic exercise likely results in little to no difference in arterial stiffness compared with land-based exercise and may be more effective for improving endothelial function, although this latter finding is based on low-certainty evidence and high heterogeneity.”

This language has been added to lines 596 (and in lines 597-599 which was already changed based on the earlier comment #1)
